# Rational Passivation of Sulfur Vacancy Defects in Two-Dimensional Transition Metal Dichalcogenides

**DOI:** 10.1021/acsnano.1c01220

**Published:** 2021-05-13

**Authors:** Hope Bretscher, Zhaojun Li, James Xiao, Diana Yuan Qiu, Sivan Refaely-Abramson, Jack A. Alexander-Webber, Arelo Tanoh, Ye Fan, Géraud Delport, Cyan A. Williams, Samuel D. Stranks, Stephan Hofmann, Jeffrey B. Neaton, Steven G. Louie, Akshay Rao

**Affiliations:** † University of Cambridge, Cambridge, CB2 1TN, U.K.; ‡ Uppsala University, Uppsala, 751 20, Sweden; ¶ Yale University, New Haven, Connecticut 06520, United States; § Weizmann Institute of Science, Rehovot, 76100, Israel; ∥ University of California Berkeley, Berkeley, California 94720, United States; ⊥ Lawrence Berkeley National Laboratory, Berkeley, California 94720, United States

**Keywords:** 2D materials, defects, spectroscopy, many-body perturbation theory, defect engineering, TMDC

## Abstract

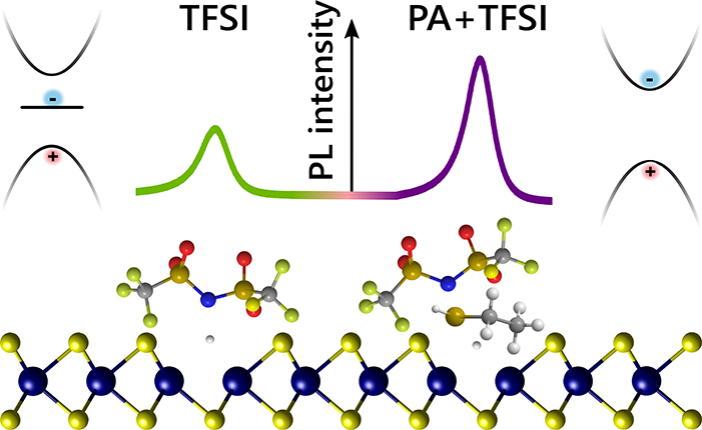

Structural defects vary the optoelectronic properties of monolayer transition metal dichalcogenides, leading to concerted efforts to control defect type and density *via* materials growth or postgrowth passivation. Here, we explore a simple chemical treatment that allows on–off switching of low-lying, defect-localized exciton states, leading to tunable emission properties. Using steady-state and ultrafast optical spectroscopy, supported by *ab initio* calculations, we show that passivation of sulfur vacancy defects, which act as exciton traps in monolayer MoS_2_ and WS_2_, allows for controllable and improved mobilities and an increase in photoluminescence up to 275-fold, more than twice the value achieved by other chemical treatments. Our findings suggest a route for simple and rational defect engineering strategies for tunable and switchable electronic and excitonic properties through passivation.

Single and few-layer semiconducting, two-dimensional transition metal dichalcogenides (2D-TMDCs) have received significant attention in recent years due to their unique optoelectronic properties. Monolayer TMDCs possess a direct bandgap, optical excitations in the visible range, and very high absorption coefficients.^1−3^ However, the photoluminescence (PL) arising from exciton radiative recombination typically shows low PL quantum yields (PLQYs); in monolayer MoS_2_ and WS_2_, prepared *via* exfoliation or chemical vapor deposition, the PLQY has been measured to be below 1% for MoS_2_ and only slightly higher for WS_2_.^1,4,5^ This low PLQY is attributed to the presence of defects in these materials,^6−9^ which quench photoluminescence and limit carrier mobilities.^5,6,10−12^ A systematic understanding of the nature of defects and the corresponding development of appropriate defect passivation strategies is hence greatly desired and is expected to improve device applications ranging from light-emitting diodes and photovoltaics to quantum emitters and future quantum information devices.

Vast recent research is dedicated to identifying defect types and their effect on material functionality in monolayer TMDCs.^9,13−20^ While numerous prototypical structural defects are considered abundant, point chalcogen vacancies have been shown to have the lowest formation energy.^21−23^ Transmission electron microscopy (TEM) and scanning tunneling microscopy (STM) have been used to identify sulfur vacancies in MoS_2_ and WS_2_, with densities of ∼10^13^ cm^–2^.^10,23−25^ These vacancies form electronic states within the bandgap, leading to the formation of low-lying defect excitons, which also form hybridized states with higher energy, band edge excitons.^18^ Recent research combining STS, atomic force microscopy (AFM), and *ab initio* theory suggests that oxygen substitutions at the chalcogen site—which appear nearly identical to chalcogen vacancies in TEM and AFM—are also abundant in TMDCs and are formed during both crystal growth and exposure to ambient conditions.^16,17,26^ Oxygen substitutions of this kind are not expected to possess the in-gap defect states or subgap excitonic states associated with the vacancy defect,^16−18,22−24,26−29^ and their impact on the optical signatures remains unclear. This microscopic understanding of the electronic and excitonic states and their relation to the structure of defects suggests possible passivation pathways to control the PLQY through defect design.

A number of methods have been proposed to increase the PLQY in TMDC samples through defect passivation, including chemical treatments^30−33^ and thermal annealing.^34,35^ Despite these efforts, the magnitude of the PL increase due to such treatments is still not satisfying and typically less than a 10-fold enhancement.^29,32,33,36−38^ A relatively successful chemical treatment for defect passivation is based on the organic superacid bis(trifluoromethane)sulfonimide (H-TFSI), which is found to increase the PL signal by orders of magnitude.^4^ Both this superacid treatment^4^ and electrical gating in a capacitive structure^39^ increase PL *via* the same pathway, namely, by reducing the high n-doping often found in both exfoliated and grown MoS_2_ and WS_2_ monolayers.^26,40−42^ These excess charges readily form trions, for which radiative recombination is much less efficient than for the neutral exciton,^29,43,44^ suggesting that excess n-doping may be the primary cause of low PLQY in these monolayers. It has also been shown that the PL lifetime increases upon TFSI treatment,^45−47^ which is not ideal for devices, as it opens up competing channels of nonradiative decay processes. In addition, carrier mobilities are shown to be limited in TFSI-treated samples, compared to as-exfoliated samples.^47^ These observations suggest that while they enhance the PL yield, such treatment may not passivate defect sites and hence does not improve defect trap-limited properties.

In this article, we explore the effect of defect passivation on PL yield and exciton lifetimes in monolayer TMDCs. We study the nature of defect states created upon various chemical passivation methods through steady-state and ultrafast spectroscopy, supported by *ab initio* GW and Bethe Salpeter equation (GW-BSE) calculations.^48−51^ We experimentally demonstrate the formation of subgap absorption features that are consistent with theoretically predicted energies for confined excitons associated with sulfur vacancies. These subgap states act as traps and lengthen PL lifetimes while limiting PL yields and carrier mobilities. We further develop a generalizable passivation protocol for sulfur vacancies, by treating samples with a passivating agent (PA), such as a thiol or sulfide, followed by a Lewis acid, such as the TFSI superacid to remove excess electrons. This two-step passivation treatment greatly enhances the measured PL, by over 275-fold from the brightest spot on untreated samples compared to the brightest spot on treated samples, and exhibits a mean enhancement of ∼10× (Supporting Information, Table 1). The treatment further decreases the PL lifetime and improves carrier mobilities by 2 orders of magnitude relative to TFSI treatment alone, suggesting passivation of sulfur vacancies. The generalizability of this protocol, which can be performed with a number of chemical agents, not only enhances the optoelectronic properties of TMDCs through passivation, but can be used to functionalize these materials and tune their PL properties.

## Results and Discussion

**Figure 1:**
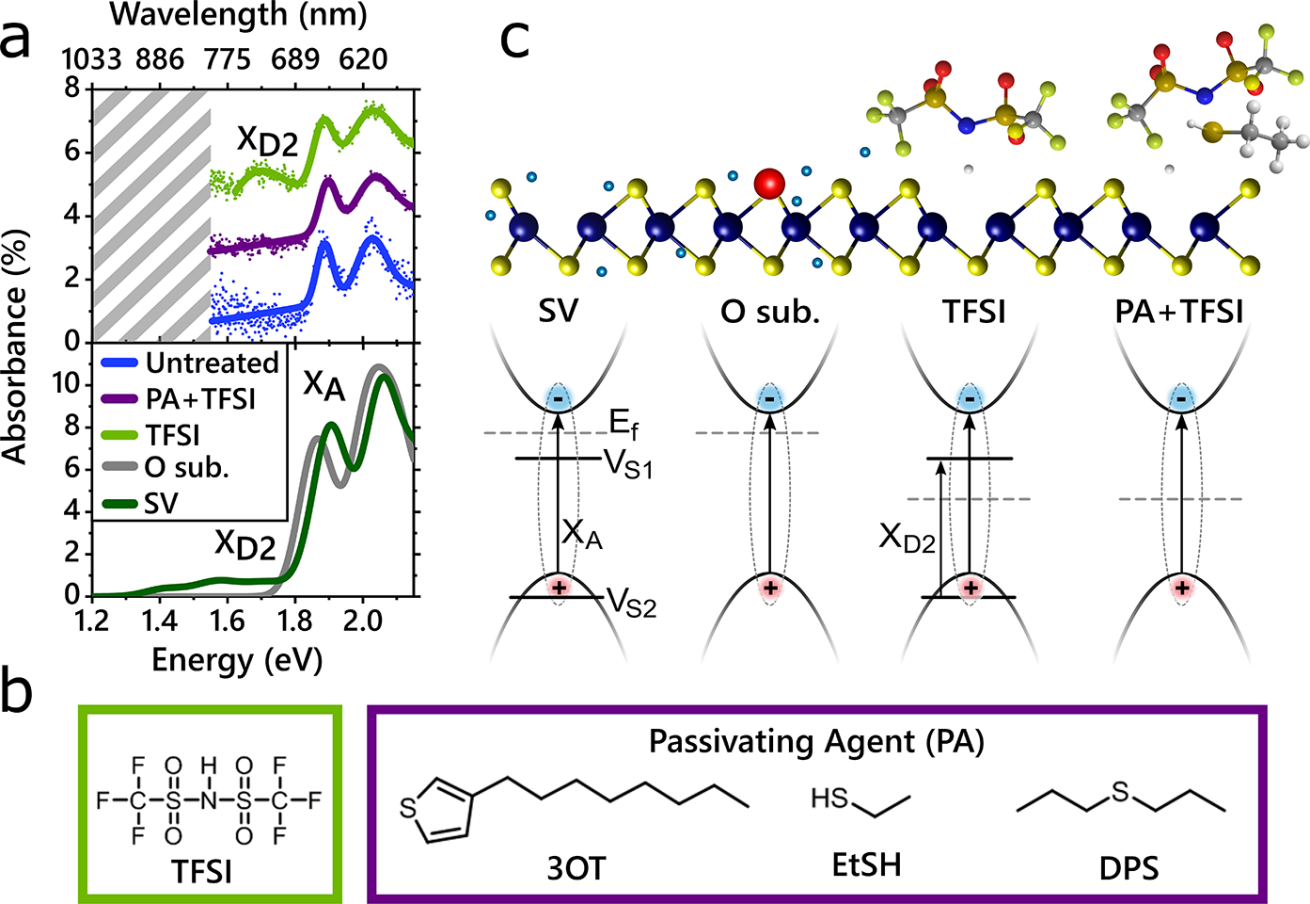
Absorption and treatment schematic. (a) Top: Absorption of untreated (blue), TFSI-treated (green), and PA+TFSI treated (purple) monolayer MoS_2_. TFSI-treated samples possess a subgap absorption peak, *X*_D2_. Bottom: Calculated *ab initio* GW-BSE absorbance spectrum of monolayer MoS_2_ with a 2% sulfur vacancy density (green line) and a 2% oxygen substitution density (gray line). *X*_D2_ corresponds to excitons arising from transitions between a defect level within the valence band (V_S2_) and an in-gap defect level (V_S1_). (b) Chemical makeup of TFSI and the passivating agents used in this study. (c) Ball-and-stick model (top) and schematic of the low-energy band structure in the K valley in MoS_2_ and proposed impact of different chemical treatments outlined in (b). MoS_2_ ball-and-stick model originally drawn in VESTA.^52^ Untreated S vacancy (SV) and untreated O substitution (O sub.) do not possess an optically accessible subgap state. In the untreated S vacancy case, the transition between the defect states (*X*_D2_) is prohibited, as the subgap state is occupied due to the high Fermi level. With an oxygen substitution, there is no in-gap defect state. TFSI treatment lowers the Fermi level and may remove an oxygen substitution, which allows the defect-to-defect transition, *X*_D2_, to occur. With PA+TFSI treatment, the Fermi level is lowered and the subgap state is eliminated.

We begin by comparing the photophysical properties of as-exfoliated (referred to as untreated) MoS_2_, which is considered to be heavily n-doped,^26,39−42^ with a sample that was chemically treated with TFSI, where the n-doping has been significantly reduced (referred to as TFSI-treated). Samples are mechanically exfoliated onto Si/SiO_2_ or fused silica substrates using the gold-assisted exfoliation method.^53^ All measurements were performed at room temperature. Figure 1a, top, compares the steady-state optical absorption spectra of untreated MoS_2_, as well as TFSI-treated spectra with and without PAs such as ethanethiol (EtSH), shown in Figure 1b. All samples possess the characteristic spin–orbit split A and B exciton peaks at ∼1.9 and ∼2.0 eV, respectively. However, in the TFSI-treated sample, a subgap absorption feature appears around 1.7 eV. The experimentally measured absorption compares well to our computed GW-BSE absorbance, shown in Figure 1a, bottom. The absorbance was calculated for a freestanding MoS_2_ monolayer with a uniform 2% concentration of sulfur vacancies and for the same system but with an oxygen substitution at the defect site. As shown previously, the sulfur vacancies give rise to a subgap feature (*X*_D2_), arising from electron–hole transitions between the defect states.^18^ The computed excitation energy associated with these transitions is in good agreement with the subgap features observed in experiment. In stark contrast, the calculated absorbance of the oxygen substitution system does not show any subgap states. This comparison to theory suggests that, surprisingly, the TFSI treatment opens up SV sites.

Figure 1c presents a schematic overview of the quasiparticle bands associated with various defect configurations near the K and K′ valleys in monolayer MoS_2_. The sulfur vacancy introduces an occupied defect state close to the valence band edge and an unoccupied defect state deep in the bandgap.^18,54^ As we show below, these unoccupied states act as traps for excitons. According to our calculations, oxygen substitutions, which previously were shown to remove the subgap defect states,^16,26,34^ also do not introduce subgap features in the optical absorption spectrum. A possible route by which the TFSI can open up the sulfur vacancy defect site, as demonstrated schematically in Figure 1c, is by removing substituted oxygen atoms from the untreated sample. Alternatively, charges from the dopants might shift the Fermi level, occupying the subgap defect states and rendering them inaccessible to optical excitations. The reduction in n-doping *via* the TFSI treatment (consistent with previous results and field-effect transistor (FET) measurements shown below) would then make these sites accessible.

**Figure 2:**
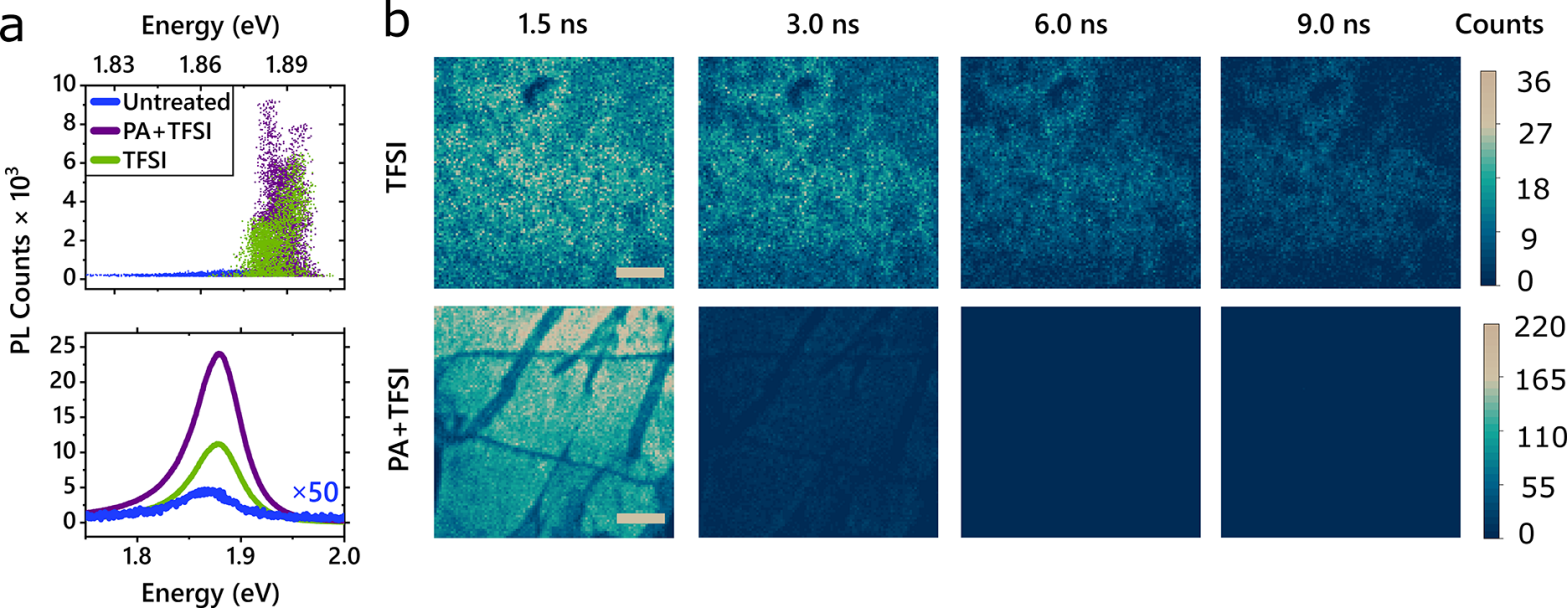
PL dynamics. (a) Top: Energy (*x*-axis) *versus* maximum PL count (*y*-axis) for samples of each treatment condition, where each point represents a pixel of a PL map. TFSI and PA+TFSI treatments result in a blue-shift and narrowing of the peak emission energy distribution. Mean and standard deviation are shown in Supporting Information, Table 1. Bottom: Sample PL spectra illustrating the brightest spot measured under each treatment condition. With optimization, the PA+TFSI chemical treatment enhances the PL about twice as much as TFSI alone. (b) PL maps illustrating the PL magnitude and intensity as a function of time after excitation, for TFSI-treated samples (top) and PA+TFSI-treated samples (bottom). Scale bar corresponds to 4 μm. Emission of untreated samples was instrument-response-limited and thus shorter than 100 ps (see Supporting Information, Section 7).

In agreement with prior studies, we observe a large enhancement in PL for TFSI-treated samples, as shown in Figure 2. The line width narrows by ∼0.06 eV and the peak emission energy blue-shifts, as shown in Figure 2a (and further statistics and data shown in the Supporting Information, Section 1). Representative measurements of the PL emission line shape and intensity are shown in Figure 2a, bottom. In Figure 2b, we measured the PL intensity as a function of time and space for TFSI (top)- and PA+TFSI (bottom)-treated flakes. The PL emission lifetime for TFSI-treated samples is ∼3.5 ns, with a standard deviation of 1.5 ns. The distribution of the average PL lifetime is between 1 and 20 ns, much longer than the PL lifetime of untreated MoS_2_,^55,56^ which falls below the instrument response of 100 ps (see Supporting Information, Section 7). The observed longer PL lifetime upon TFSI treatment is consistent with previous results and has been suggested to arise due to a trap-mediated exciton recombination process following TFSI treatment.^45,57^ We note that in the absence of defects shorter radiative exciton lifetimes are expected (based on their high absorption coefficients), allowing emission to outcompete nonradiative decay channels.^55^

**Figure 3:**
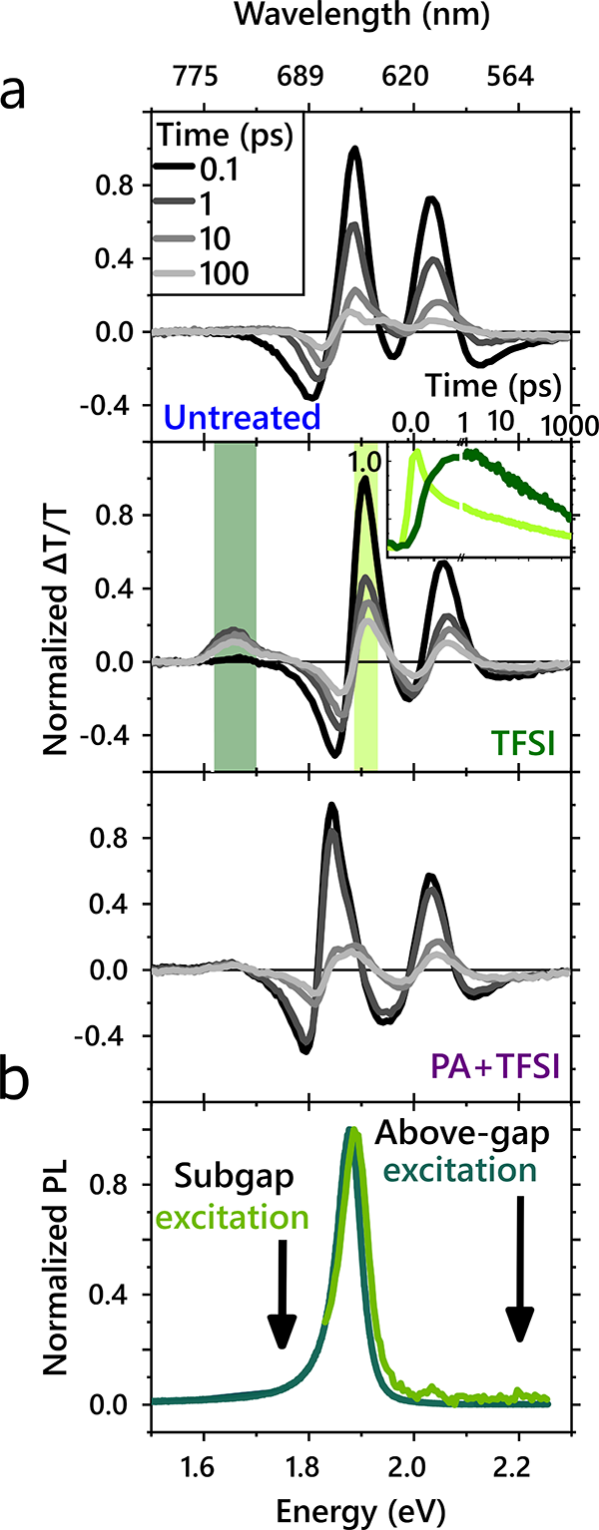
(a)Pump–probe measurements of MoS_2_ monolayers with different chemical treatments. Pump–probe spectra of MoS_2_ untreated (top), TFSI-treated (middle), and PA+TFSI treated (bottom) MoS_2_. TFSI treatment results in a prominent subgap bleach associated with sulfur vacancy defects. The inset shows the normalized kinetics taken at the A exciton bleach (light green) and defect peak (dark green) in the TFSI-treated sample, illustrating transfer from the band edge to the subgap defect state. (b) PL emitted with subgap excitation of TFSI-treated MoS_2_ occurs at the same energy as PL emitted by above-bandgap excitation of TFSI-treated MoS_2_.

To further study the energy-resolved time evolution of the excited state in MoS_2_, we performed ultrafast pump–probe measurements on monolayer flakes >150 μm in diameter (measurements were performed on multiple samples to ensure reproducibility; see Experimental Section). In these measurements, samples are excited with a narrowband, close-to-resonant pump pulse at 1.92 eV and probed using a broadband white-light pulse. As shown in Figure 3, both the untreated and TFSI-treated samples possess positive features around 2.03 and 1.88 eV. As the change in transmission is proportional to the change in the density of states, we assign these positive features to the bleach of the A and B excitons. When the A and B exciton states are populated, fewer photons are absorbed from the probe pulse, resulting in a positive signal in the differential pump–probe measurement.^58^

In the TFSI-treated sample, Figure 3a, middle, a positive feature in the near-IR appears at the same energy as the state previously observed in the steady-state absorption measurements (Figure 1a, top). We attribute this feature to a bleach of the subgap defect state. This defect state is lower in energy and delocalized in momentum space, so excitons could be expected to funnel into these states. Indeed, we observe that the bleach of the A exciton in the TFSI-treated sample decays more significantly than the bleach of the untreated A exciton in the first 50 ps (Figure 3a, middle, inset, and Supporting Information Section 2). Simultaneous with the initial A exciton decay (light green) is a growth of the subgap defect bleach (dark green), confirming a transfer in population from the band edge A excitons to the defect states. As the photoexcited excitons localize in these low-energy states, absorption into those states is reduced, thereby increasing the bleach signal. The hybridization of states is further corroborated by pump–probe measurements with pump energies resonant with the defect states (see Supporting Information, Section 4). While TFSI-treated samples show clear bleach signatures from the A and B excitons from time zero, untreated samples exhibit negligible signal for all times, illustrating that the untreated samples do not absorb the lower energy pump.

When excited above the bandgap, the emission from TFSI-treated samples is dominated by photons near the optical band edge, as shown in Figure 3b, dark green. Surprisingly, with subgap excitation, resonant with the defect-state absorption at 1.70 eV, we also observe emission from the band edge at 1.88 eV (Figure 3b, light green). Although the pump–probe measurements illustrate that carriers populate the subgap state, the emission does not occur at this energy. This is consistent with previous reports of temperature-dependent PL, suggesting that thermally activated upconversion from trap states in TFSI-treated MoS_2_ may be responsible for the enhanced PL.^45^ Interestingly, based on our observations, upconversion from the defect state to the A exciton state must happen on faster time scales than the radiative and nonradiative decay of the defect exciton, for which previous works have measured long emission lifetimes.^45,59,60^ As discussed in the Supporting Information Section 8, we estimate, based on the Raman modes of the system, that thermal repopulation of the A exciton is possible on a time scale of hundreds of picoseconds to a nanosecond. This gives us a lower bound on the defect exciton radiative and nonradiative decay rates, as both must be slower than the repopulation to the A exciton.

**Figure 4:**
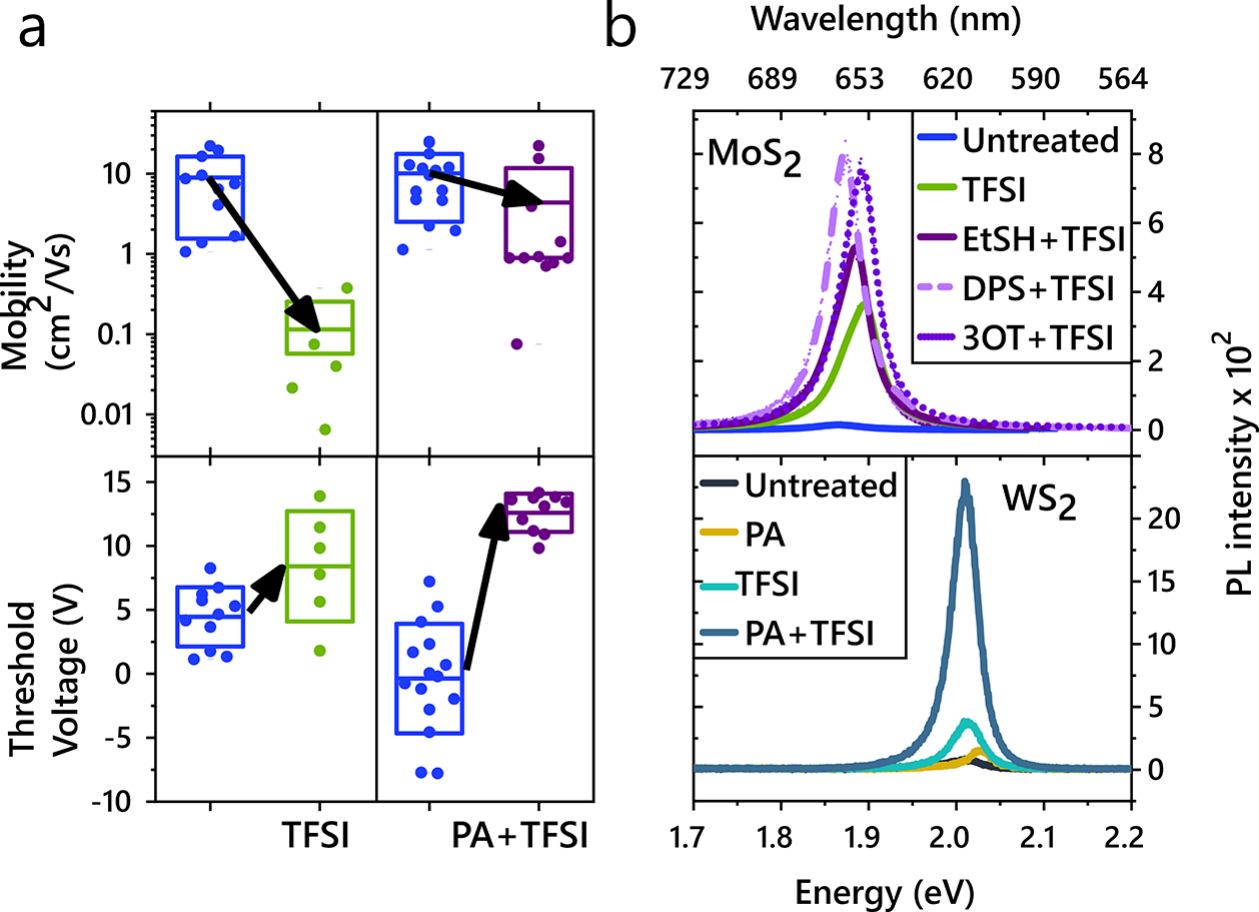
Device properties and generalizability of chemical treatment. (a) Field-effect mobility (top) and threshold voltage shift (bottom) for FET devices before and after treatment, plotting the mean (solid line) and standard deviation (box) of measurements on different devices. Although both TFSI and PA+TFSI treatment decrease the threshold voltage, the PA+TFSI treatment maintains the mobility, whereas TFSI alone results in a significant reduction in mobility. (b) Top: PL enhancement of MoS_2_ using the different passivating agents outlined in Figure 1b. Bottom: PL enhancement of various chemical treatments on WS_2_. PA alone results in a very minimal increase in PL (also see Supporting Information Section 6).

Thus, we find that, at room temperature, TFSI treatment enhances emission, rather than decreasing it. Emission occurs in spite of the presence of sulfur-vacancy-related subgap states, which trap excitons and prolong the PL lifetime. If these decay routes were faster than the repopulation of the A exciton, emission from the A exciton would be quenched as this defect-decay route would be the dominant process. At room temperature, there is sufficient energy to thermalize to the band edge, where excitons emit. Because the emission comes primarily from the neutral exciton, the line width narrows and blue-shifts with respect to untreated samples where trion emission may lead to broadening.^61^ However, despite the increase in PL emission, we observed a decrease in free-carrier mobility by over 2 orders of magnitude in TFSI-treated FETs as compared to untreated FETs, shown in Figure 4a. So while TFSI may increase the PLQY, the SVs still trap carriers and play a significant role in the dynamics. This implies that the defects remain a barrier and significantly limit the quality of devices. Therefore, we seek to determine routes to passivate defects, as discussed below.

We aim to understand the passivation mechanism in order to rationally design chemical protocols to control defect nature and dynamics. Recent theoretical predictions suggest that a thiol bound to the SV in MoS_2_ would push the site energy above the bandgap, hence effectively passivating the SV.^62,63^ Previous studies attempting to use thiol groups to passivate SVs in TMDCs have found them to be ineffective, resulting in a reduction in PL.^64^ We speculate that this is because thiols are a minor n-dopant.^65,66^ When applied to MoS_2_, though they may passivate defects, they add additional carriers and reduce the spectral weight of the neutral exciton, which limits the quantum yield.^61^ We therefore treat samples with a combination of a passivating agent, which we show below can be from a range of different chemicals, and a strong Lewis acid, TFSI (Figure 4b, top, Supporting Information, Section 6).

The maximum PL spectra observed on a PA+TFSI-treated sample is ∼275 times brighter than the brightest point on untreated samples (Figure 2a, bottom). The PL enhancement obtained by the two-step treatment is on average twice as large as for the TFSI-only treatment, as shown in Figure 2a, with further figures and statistics reported in the Supporting Information, Section 1. Similar to the TFSI-treated samples, the peak PL position blue-shifts by roughly 30 meV, consistent with a decrease in trions. We illustrate the increased PL intensity and shortened emission lifetime through color maps, plotting the PL counts at different times after excitation, shown in Figure 2b, bottom. The PL is both more intense and shorter in lifetime for the PA+TFSI sample than the TFSI-only sample. Further statistics comparing multiple flakes of each treatment condition are found in the Supporting Information, Section 1. The lifetime has a mean of 2.5 ns with a standard deviation of 1 ns (Supporting Information Table 2), shorter than the mean lifetime of TFSI-treated flakes, of 3.5 ns. Theoretical predictions of the PL lifetime for MoS_2_ at room temperature are in the 500 ps to few-nanosecond time range.^55,56^ Thus, the lower lifetimes obtained *via* the two-step treatment are more in line with what would be expected from the intrinsic, non-trap-limited PL decay. Turning to the steady-state absorption (Figure 1a, top), no subgap states are observed for the two-step treatment, in contrast to the TFSI treatment. Correspondingly, in our pump–probe measurements (Figure 3a, bottom), we observe a greatly reduced subgap bleach, which as discussed above arises from the subgap SV sites. This suggests that the two-step treatment greatly passivates these SV sites, while increasing PLQY above that achieved *via* TFSI-only treatment. Further optimization on these treatments should allow for complete passivation of the SV sites, resulting in higher PL yield and shorter, more intrinsic lifetimes.

The reduction in defect-trap-limited dynamics is further illustrated by FET measurements, establishing the free carrier mobility for each treatment condition. The field-effect mobility of TFSI-treated samples decreased by 2 orders of magnitude upon treatment (Figure 4a, top). This could be partially due to a decrease in doping, as seen also in the threshold voltage shift (Figure 4a, bottom). However, PA+TFSI-treated samples exhibit field-effect mobilities of the same order of magnitude as untreated devices, despite a threshold voltage change comparable to TFSI-treated samples. When the carrier density is comparable to the charge trap density, a significant proportion of the carriers are localized, deteriorating the charge carrier mobility.^25^ As such, experimental reports typically observe mobility in MoS_2_ increasing with increasing carrier density. The reduced field-effect mobility we observe in TFSI is therefore expected due to the decreased carrier density, determined from a positive threshold voltage shift, approaching the charge trap density. In contrast, devices with PA+TFSI treatment exhibit a comparable positive threshold voltage shift but without a significant reduction in field-effect mobility, indicating a reduction in the charge trap density relative to TFSI treatment alone. We therefore attribute this decrease in mobility in TFSI-treated MoS_2_ to the prevalence of subgap SV sites, which can be greatly passivated by the two-step treatment developed here, thereby increasing mobilities in PA+TFSI-treated samples relative to TFSI treatment alone.

We conclude by discussing the generalizability of the treatment methods presented in this work. First, in Figure 4b, top, we show that the initial passivation step is achievable using a range of chemicals with sulfur in the −2 oxidation state, such as sulfides and thiols. Second, to confirm that our results can be generalized to other sulfur-based TMDCs, we illustrate similar PL enhancements on WS_2_ shown in Figure 4b, bottom. Thus, guided by the understanding of defects in these materials, we are able to design a passivation protocol for reducing SV defects. We illustrate that PLQY alone is insufficient to determine material quality in 2D materials, as PL can significantly increase even in the presence of sulfur vacancy defects. Our study of the absorption and PL lifetime upon different chemical treatments in MoS_2_ and WS_2_ highlights the importance of designing mechanistic chemical treatments to reduce the influence of defects and increase the material functionality.

## Conclusions

These protocols, however, are not limited solely to passivation of defects. Instead, we identify a treatment scheme for defect engineering of TMDCs. By tuning material properties *via* a simple, solution-based method, such rational passivation strategies can allow for defects to be used as a handle, rather than a hindrance. Along with improvements in properties like PL and mobility, we identify a possible design rule for effective treatments and a wider range of chemicals with −2 sulfur as an effective defect engineering route for specific functionality or to break a symmetry,^67^ which is beyond the scope of this work. These chemical passivation routes can also be easily translated into device manufacturing, allowing for both improved devices and device capabilities.

## Experimental Section

### Sample Preparation

Samples are exfoliated from bulk MoS_2_ and WS_2_ from 2DSemiconductors following the gold-evaporation method previously reported,^53^ onto either quartz or Si/SiO_2_ substrates. Further information on their sample quality is shown in the Supporting Information, Section 3.

### Chemicals

All solvents used in chemical treatments were anhydrous and purchased from Sigma-Aldrich. 3-*n*-Octylthiophene was purchased from Tokyo Chemical Industry UK, Ltd., and used directly. Other chemicals used in these treatments were purchased from Sigma-Aldrich and used as received.

### TFSI Preparation and Treatment

Three different concentrations of TFSI solutions were used in this work. TFSI solutions of 0.02 and 2 mg/mL were prepared according to the literature.^4^ A 5 mg/mL (∼0.018 M) TFSI solution was prepared in a nitrogen glovebox by dissolving 100 mg of TFSI in 20 mL of 1,2-dichlorethane (DCE). The PL intensity of TFSI-treated MoS_2_ and WS_2_ increased while increasing the concentration of TFSI, until 5 mg/mL, after which it saturated. Samples were submerged in TFSI solution for ∼30 min, then dried with a nitrogen gun. The TFSI solution could also be drop casted. We found no significant difference between these two methods. All the preparations of chemical solutions and chemical treatments were carried out in a nitrogen glovebox.

### Ethanethiol Preparation and Treatment

The 0.1 M ethanethiol solution was prepared by mixing 14.8 μL of ethanethiol with 20 mL of acetonitrile. A 0.1 M ethanethiol/acetonitrile mixture was spin coated on the TMDC sample at 1000 rpm for 1 min. The sample was rinsed in toluene to remove extra ethanethiol that was not chemisorbed on the TMDC surface and was dried with a N_2_ gun. Finally, the sample was placed on a hot plate at 100 °C for 5 min. We found that the annealing step had no impact on optical and electronic properties of samples and was used to decrease the drying time. The ethanethiol was also compatible with dichloromethane and 1,2-dichloroethane.

### Dipropyl Sulfide and 3-*n*-Octylthiophene Preparation and Treatment

The 0.1 M dipropyl sulfide solution was prepared by mixing 282 μL of dipropyl sulfide with 20 mL of dichloromethane. The 0.1 M 3-*n*-octylthiophene solution was prepared by mixing 427 μL of 3-*n*-octylthiophene with 20 mL of dichloromethane. Samples were submerged in 4 mL of chemical solutions in a 25 mL glass vial overnight, after which the samples were rinsed in dichloromethane and dried with a nitrogen gun.

### Absorption

Steady-state absorption measurements were performed in transmission on a Zeiss Axiovert inverted microscope with a halogen white light source and a Zeiss EC Epiplan Apochromat 50× objective (numerical aperture (NA) = 0.95). The light transmitted through the sample is coupled to a UV600 nm optical fiber connected to a spectrometer (AvaspecHS2048, Avantes).

### Steady-State PL Measurements

Multiple experimental setups were used to measure photoluminescence; however data that are displayed on the same figure is always measured on the same setup under the same conditions.

In a WITec Alpha 300 s setup,^68^ a 405 nm continuous wave laser (Coherent CUBE) is fiber coupled into a microscope with a 20× Olympus lens to excite the sample. The emitted PL is collected through the same 20× objective, then sent to a Princeton Instruments SP-2300i spectrometer and an Andor iDus 401 CCD detector. All PL is measured at 30 W cm^–2^. Data from this setup are shown in Figure 4b, bottom, and Supporting Information, Section 1.

A second setup is used for measurements where untreated MoS_2_ is directly compared to chemically treated samples (Figure 2a, Supporting Information Section 6), and Figure 4b, top). This is performed on a Renishaw inVia confocal setup, excited with a 514.5 Ar-ion continuous wave laser, exciting with a 20× objective and ∼30 W cm^–2^. The PL is collected in reflection and dispersed using a 600 L/mm grating onto a CCD detector.

The upconversion PL measurement (Figure 3b) is performed on a home-built linear PL setup. For above-bandgap excitation, we use a 405 nm laser (Coherent Obis 405) exciting with ∼52 mW cm^–2^. For subgap excitation, we excite with a 730 nm diode laser (Thorlabs) with ∼2 W cm^–2^. PL is collected with a lens, focused into an Andor Kymera 328i spectrometer, and then recorded with a Si-CCD (Andor iDus 420). In the subgap excitation measurement, a short-pass filter at 700 nm is used to remove the laser, though a small tail is still observed.

Further PL measurements in the Supporting Information, Section 3 are carried out on a Renishaw inVia Raman confocal microscope with a 532 nm excitation laser in air under ambient conditions. The Raman emission was collected by a 50× long working distance objective lens in streamline mode and dispersed by a 1800 l/mm grating with 1% of the laser power (<10 μW). The spectrometer was calibrated to a silicon reference sample prior to the measurement to correct for the instrument response.

### Time-Resolved PL Measurements

Time-resolved measurements are performed using a PicoQuant Microtime 200 confocal time-resolved PL setup. Samples are excited with a 405 nm, 20 MHz pulsed laser focused by a 20× objective (0.6 Numerical Aperture), and emitted PL is collected in reflection mode and sent to a hybrid photomultiplier tube detector from Picoquant for single-photon counting. The emission signal was separated from the excitation light (405 nm) using a dichroic mirror (Z405RDC, Chroma). On the emission path, a pinhole (50 μm) is used for the spatial filtering, as well as an additional 410 nm long-pass filter. Repetition rates of 27 MHz were used for the maps. The instrument response is measured to be ∼100 ps (Supporting Information Section 7); 100 nW of power is used to excite a ∼ 2.5 × 2.5 μm^2^ area, corresponding to a 80 nJ cm^–2^ fluence. At each point, the PL intensity and time-resolved photoluminescence decay curve are measured. The PL counts are then binned into four time intervals, 0–1.5, 1.5–3, 3–6, and 6–9 ns. This binning of data is then plotted in Figure 2b. Every spatial data point can also be thought of as a distribution of photons arriving at different delays after the excitation pulse, also called a photon delay histogram. This allows us to evaluate the PL lifetime, by measuring the weighted average of the photon histogram at each point. Both the PL intensity and the PL decays are plotted in a scatter plot shown in the Supporting Information, Figure 1, bottom. Untreated samples have a short decay lifetime, <100 ps, as the experimentally measured PL lifetime is instrument-response limited (Supporting Information Section 7). Finally, the statistics from these data are shown in Supporting Information, Table 1.

### Pump–Probe

Pump–probe measurements are performed using a Light Conversion Pharos Yb-based system, with 400 μJ per pulse at 1030 nm and a repetition rate of 38 kHz. The output of the Pharos is split and sent along two paths, one to generate the pump and the second the probe. In the pump path, the fundamental of the laser is sent into a narrow-band optical parametric oscillator system (ORPHEUS-LYRA, Light Conversion), where ∼250 fs fwhm pulses are generated, for most measurements in this work, at 500 nm. The pump is chopped with a mechanical chopper to generate an on–off pattern with respect to the probe pulse. The probe is generated by focusing the fundamental of the laser into a 4 mm YAG crystal, producing a broadband probe from 520 to 950 nm. The probe is delayed with respect to the pump, using a computer-controlled mechanical delay stage (Newport). The probe, of roughly 80 × 80 μm^2^ in spot size, is kept intentionally smaller than the ∼250 × 250 μm^2^ pump. The monolayer is found by centering a ∼200 μm^2^ diameter onto the substrate when under a microscope, then aligning the probe through the center of this pinhole. The detected probe is collected on a silicon line scan camera (AViiVA EM2/EM4) with a visible monochromator 550 nm blazed grating. Data shown for the untreated and PA+TFSI treatment are taken from the same sample (measured before and after treatment). These data are also representative of measurements taken over many samples for each treatment condition (>15 TFSI-treated samples, >15 untreated samples, and >5 PA+TFSI-treated samples).

### Device Preparation and Measurement

**Figure 5:**
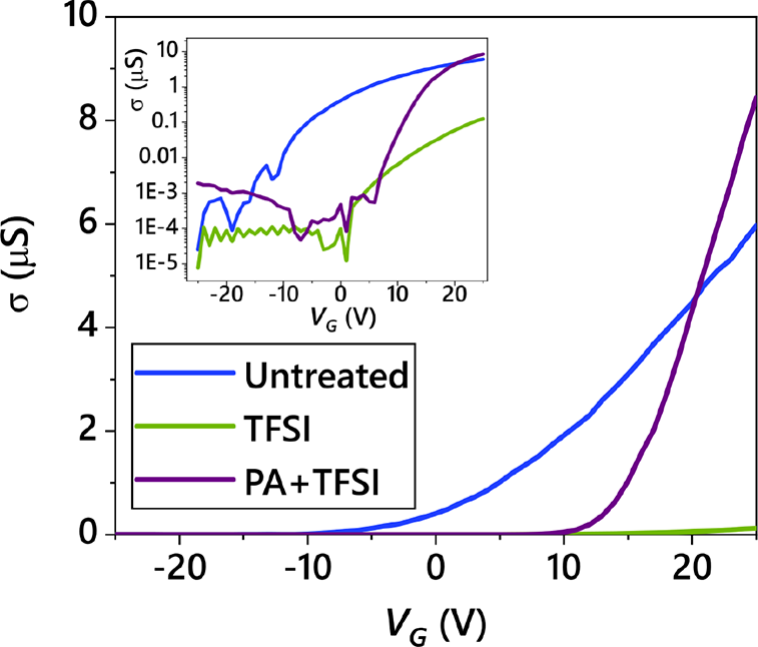
Sample transport characteristics. An example of the gate voltage *versus* mobility for different chemical treatment steps.

To determine the effects of the treatments on the electronic properties of the MoS_2_, we fabricated field-effect transistors. MoS_2_ was transferred onto Si with 90 nm of thermally grown SiO_2_, which acted as a global back gate. Monolayer MoS_2_ flakes were identified and Au contacts made by electron beam lithography and thermal evaporation. The MoS_2_ channels have a typical length of 5 μm from source to drain. Transfer characteristics were measured with a source–drain bias of *V*_DS_ = 1 V using a Keithley 4200-SCS parameter analyzer and probe station under dark ambient conditions. Example transfer curves showing the conductivity  as a function of back gate *V*_G_ for MoS_2_ devices after different treatments are plotted in Figure 5, where *L* and *W* are the length and width, respectively, and *I*_D_ is the measured current.

## Supporting Information Available

Further data and statistical analysis of PL emission enhancement and PL lifetimes; data showing the untreated samples’ PL lifetime and comparisons with the instrument response; pump–probe measurements of PA only treated samples; analysis of the kinetic lifetimes in pump–probe; PL comparison of exfoliation methods; subgap pump–probe measurements; discussion and data on varying the treatment order; discussion and calculations using the Arrhenius equation; further descriptions of the GW-BSE theory calculation; discussion on substitutional oxygen and Fermi level (PDF)

## Notes

This study is also associated with ref (75) on the preprint server Arxiv.
