# Correction to “Rational Passivation of Sulfur Vacancy Defects in Two-Dimensional Transition Metal Dichalcogenides”

**DOI:** 10.1021/acsnano.1c04628

**Published:** 2021-06-14

**Authors:** Hope Bretscher, Zhaojun Li, James Xiao, Diana Yuan Qiu, Sivan Refaely-Abramson, Jack A. Alexander-Webber, Arelo Tanoh, Ye Fan, Géraud Delport, Cyan A. Williams, Samuel D. Stranks, Stephan Hofmann, Jeffrey B. Neaton, Steven G. Louie, Akshay Rao

In the methods section, “Device Preparation and Measurement”, the caption of Figure 5 incorrectly stated, “An example of the gate voltage *versus* mobility for different chemical treatment steps.” This should instead state, “Example transfer curves showing the conductivity, σ, as a function of back gate, *V*_G_, for MoS_2_ devices after different treatments.” This is correctly described in the text referring to this figure, but the caption was mislabeled. The amended Figure 5 appears below. We apologize for the mistake in the original submission.

**Figure 5:**
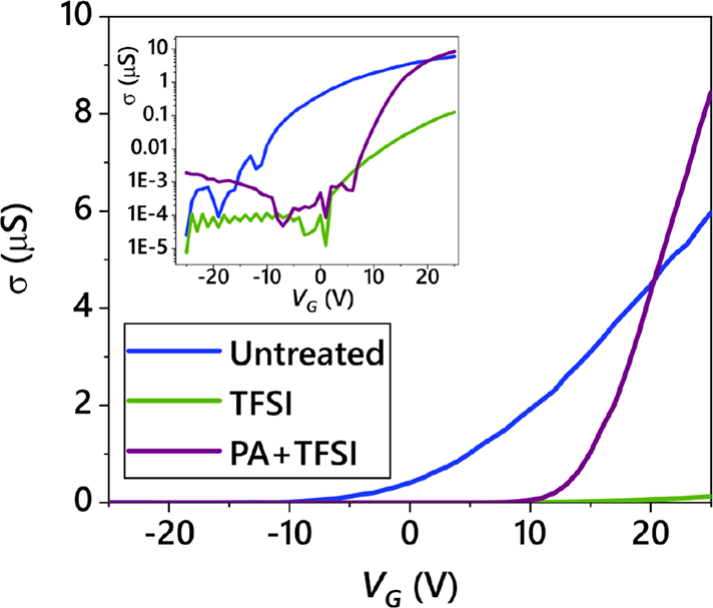
Sample transport characteristics. Example transfer curves showing the conductivity, σ, as a function of back gate, *V*_G_, for MoS_2_ devices after different treatments.

