# Supplementary material for: Rational Passivation of Sulfur Vacancy Defects in Two-Dimensional Transition Metal Dichalcogenides
Source: ACS Nano. 2021 May 13;15(5):8780–9. doi: 10.1021/acsnano.1c01220 (PMC8158852; doi:10.1021/acsnano.1c01220)
Supplement: Supplementary file 1 — nn1c01220_si_002.pdf [file nn1c01220_si_002.pdf]

Supporting Materials For: Rational Passivation of Sulfur Vacancy Defects in Two-Dimensional Transition Metal Dichalcogenides

Hope Bretscher,[†] Zhaojun Li,[†,‡] James Xiao,[†] Diana Yuan Qiu,[¶] Sivan Refaely-Abramson,[§] Jack A. Alexander-Webber,[†] Arelo Tanoh,[†] Ye Fan,[†] Géraud Delport,[†] Cyan A. Williams,[†] Samuel D. Stranks,[†] Stephan Hofmann,[†] Jeffrey B. Neaton,[‖,⊥] Steven G. Louie,[‖,⊥] and Akshay Rao[*,†]

†*University of Cambridge, Cambridge, CB2 1TN, UK*

‡*Uppsala University, Uppsala, 751 20, Sweden*

¶*Yale University, New Haven CT, 06520, USA*

§*Weizmann Institute of Science, Rehovot, 76100, Israel*

‖*University of California Berkeley, Berkeley, CA, 94720, USA.*

⊥*Lawrence Berkeley National Laboratory, Berkeley, CA, 94720, USA*

E-mail: ar525@cam.ac.uk

Section 1: PL enhancement

To gain a greater understanding of the distribution of enhancement, we acquire PL maps, measuring the intensity of PL on different samples before and after different treatment methods. Using a PL microscope, we measure a PL spectrum at each point in real-space of a monolayer flake. After subtracting off a linear background to remove any contribution from scatter and detector noise, we then determine the maximum PL counts on each spectrum and

the energy at which this occurs. This is then plotted in a scatterplot, such that every point on a monolayer flake is represented by a corresponding point on the scatterplot. We acquire maps at two different times, first before further optimization of the PA+TFSI procedure (Fig.2a,top) in the main text, and after optimization (Fig. 1top).

In Fig.2a,top, we acquire PL maps on five different samples each of untreated, TFSI, and PA+TFSI samples. The measurements were performed on a Renishaw Invivo system, which can detect the PL of untreated samples. Both TFSI and PA+TFSI samples blueshift in their peak PL energy from 1.866 eV to 1.886 eV and 1.888 eV respectively, as well as narrowing of energy distribution from 0.012 eV to 0.006 and 0.005 eV respectively. PA+TFSI shows a greater enhancement in PL counts than TFSI alone. The improvement by PA+TFSI after more optimization of the treatment procedure is further shown in Fig. 1, in which the PL enhancement by PA+TFSI is much greater than that of TFSI alone, as the mean PL counts for PA+TFSI treatment is ∼4x TFSI alone. On this measurement, performed on the WITec Alpha system, the PL of the untreated sample was undetectable due to setup sensitivity.

Table 1: Statistics from scatter plots shown in Fig. 1,top and Main text, Fig. 2a,top.

Figure	Treatment	Mean energy (eV)	Mean counts	Max counts
1b,top	TFSI	1.880 ± 0.004	123 ± 19	378
1b,top	PA+TFSI	1.880 ± 0.004	595 ± 141	837
Main text Fig. 2a,top	Pristine	1.866 ± 0.012	25 ± 6	51
Main text Fig. 2a,top	TFSI	1.886 ± 0.006	200 ± 140	651
Main text Fig. 2a,top	PA+TFSI	1.888 ± 0.005	270 ± 20	924

PL enhancement and lifetime

We make similar spatial maps to measure the PL intensity and lifetime, as shown in Fig.2a and Fig. 1,bottom. In these maps, we acquire the PL intensity as a function of time, using a bandpass filter to select the photoluminescence. In Fig. 1, we make a scatter plot for two flakes of each treatment condition (one of which is shown in the main text, Fig.2b), plotting the PL intensity and lifetime. PA+TFSI samples have a narrower distribution and shorter

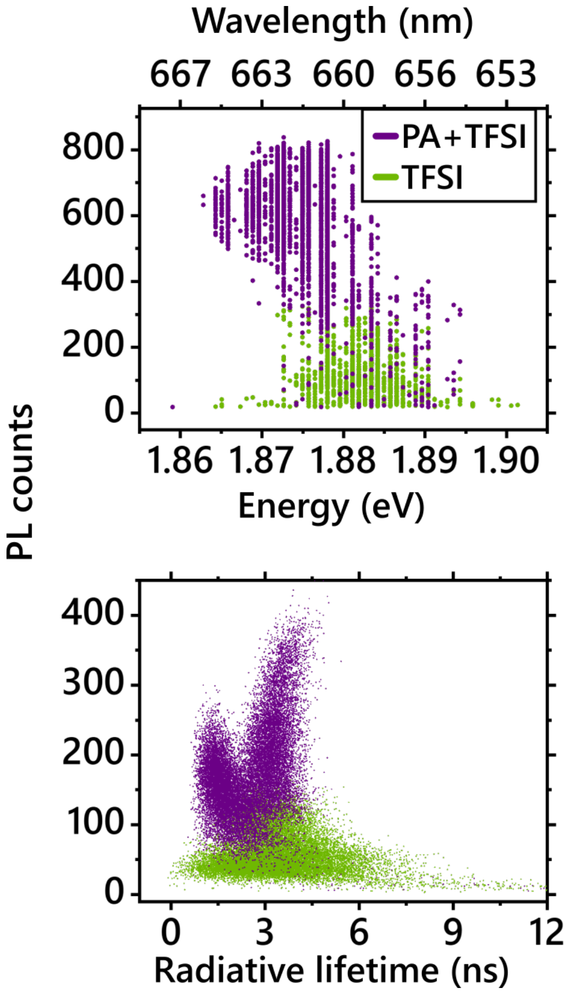

Figure 1: PL enhancement of TFSI and PA+TFSI treatment. Top: Emission energy *versus* maximum counts. With further optimization, PA+TFSI treatment shows PL >2x as bright as TFSI alone. Bottom: The emission lifetime shortens and the distribution of emission lifetimes narrows with PA+TFSI treatment as compared to TFSI only. The bimodal distribution is because data shown comes from two different samples. Statistics are shown below.

lifetime than TFSI only samples, with statistics given in Table 2.

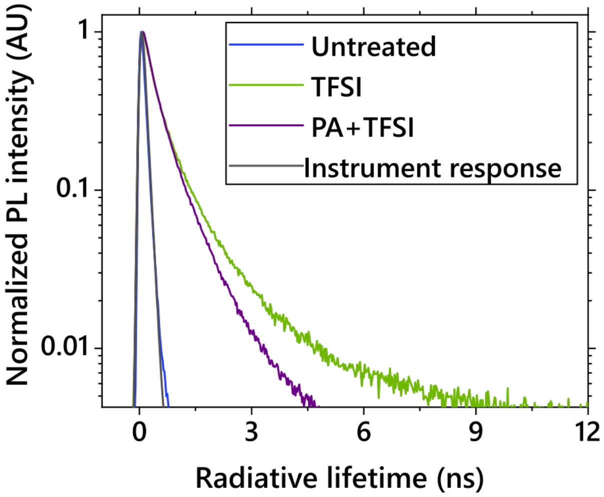

Figure 2: Illustrative time-correlated single photon counting (TCSPC) trace for untreated, TFSI, and PA+TFSI treated samples. The untreated sample's PL is indistinguishable from the instrument response (shown in grey), so we can only conclude that the lifetime is shorter than 100 ps.

Table 2: Statistics from scatter plots shown in Fig. 1,bottom

Treatment	Mean lifetime (ns)	Mean counts
TFSI	3.45 ± 1.51	60.8 ± 30.4
PA+TFSI	2.5± 1.00	179 ± 65

Section 2: Pump-probe lifetime comparisons

To further compare the dynamics of different treatment conditions, we fit the decay lifetimes of the untreated and TFSI-treated samples with an fit function where an error function (to simulate the pump-induced rise in the signal) is convolved with a series of exponential decays,

$$\frac{\Delta t}{t} = y_0 + 0.5 \times A_1 \exp(\frac{s^2}{2\tau_1^2}) \times \exp(-\frac{t-t_0}{\tau_1}) \times (1 + \mathrm{erf}(\frac{t-t_0-s^2}{\tau_1 s\sqrt{2}}))$$
$$+ 0.5 \times A_2 \exp(\frac{s^2}{2\tau_2^2}) \times \exp(-\frac{t-t_0}{\tau_2}) \times (1 + \mathrm{erf}(\frac{t-t_0-s^2}{\tau_2 s\sqrt{2}}))$$
$$+ 0.5 \times A_3 \exp(\frac{s^2}{2\tau_3^2}) \times \exp(-\frac{t-t_0}{\tau_3}) \times (1 + \mathrm{erf}(\frac{t-t_0-s^2}{\tau_3 s\sqrt{2}})) \quad (1)$$

In this equation, s is the instrument response, t_0 is time zero (when the pump and probe overlap), y_0 is a small y offset often from residual scatter, and A and τ correspond to the amplitude and decay time constant of each exponential decay. We use this equation to fit the decay kinetics for the A exciton bleach and defect bleach of the untreated and TFSI treated samples, as shown in Fig. 3, and with the corresponding fit parameters shown in table 3.

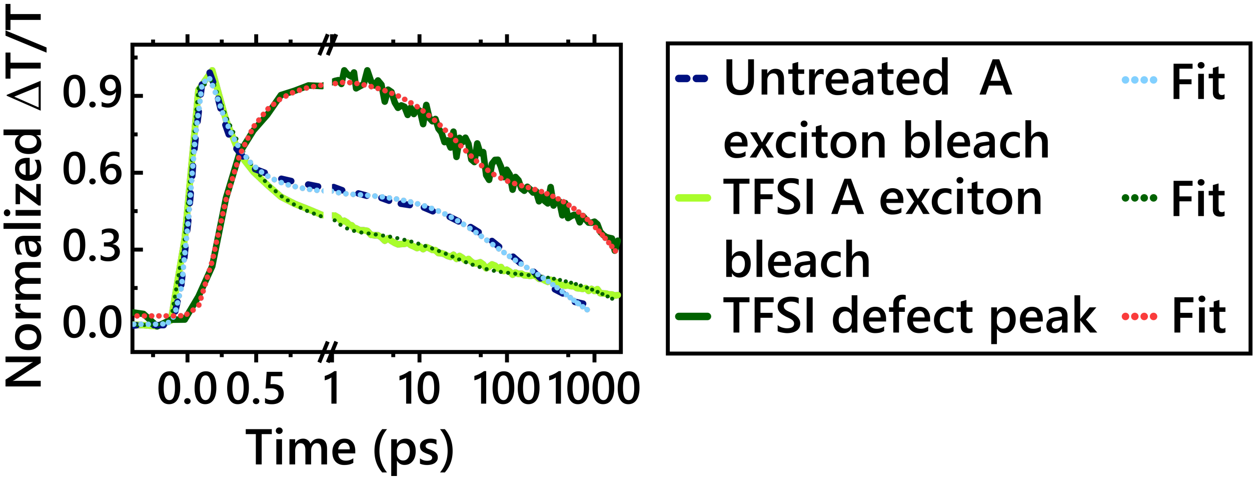

Figure 3: Pump-probe kinetics of the A exciton bleach and defect bleach. The dominant decay of the untreated sample is longer-lived than that of the TFSI treated sample. Fits are performed using equation 1

The A-exciton bleach of both the TFSI-treated sample exhibits a sub-picosecond decay component. After this initial decay, the bleach signal persists for a lifetime of tens of picoseconds. The subgap state (dark green) instead exhibits a sub-picosecond growth, before a similarly slow decay. By fitting the data with a fit function that includes an error function convolved with an exponential rise (to simulate the rise of the pulse), and a sum of exponen-

Table 3: Fit parameters using Eqn. 1 We first fit the A exciton bleach of both samples to determine the rise time (s), and then include this as a fixed parameter in the fitting process of the defect bleach. This allows us to capture the rise of the defect peak (which is not instrument response limited), as an exponential decay with a negative amplitude. All temporal units are in picoseconds.

	Untreated (bleach)	TFSI (bleach)	TFSI (defect)
A_1	0.85	0.94	-0.93
τ_1	0.21	0.34	0.21
A_2	0.21	0.16	0.39
τ_2	45.22	23.04	26.86
A_3	0.31	0.21	0.54
τ_3	490	2200	2300
Rise time (s)	0.07	0.07	0.07
Time zero (t_0)	0.03	0.02	0.12
Y offset (y_0)	0.01	0.01	0.04
χ^2_{red}	0.997	0.993	0.987

tial decays, we can give approximate time constants and amplitudes for each of these decay times (see 3 and 3). We note that the A exciton bleach of the TFSI-treated sample decays more significantly in the first 50 ps than the untreated sample. Simultaneous to its early decay is a rise of the defect state. We emphasize that these exact numbers vary to some degree sample to sample. Instead, we emphasize that this trend observed from comparing >5 untreated and TFSI treated samples.

Section 3: Exfoliation comparisons

To confirm that the effect of our chemical treatments is not due to our use of the gold exfoliation method, we compare the PL enhancement of sticky-tape exfoliated samples with those of gold-exfoliated samples, shown in Fig. 4. We exfoliate monolayer flakes using each method, and then measure the PL of the same monolayer flake before and after each treatment condition. We find that the TFSI and PA+TFSI treatments lead to significant enhancements in the original PL magnitude of each sample, confirming that our exfoliation method results in monolayers of similar quality as sticky tape exfoliated samples, as has been

previously found in previous works.? ?

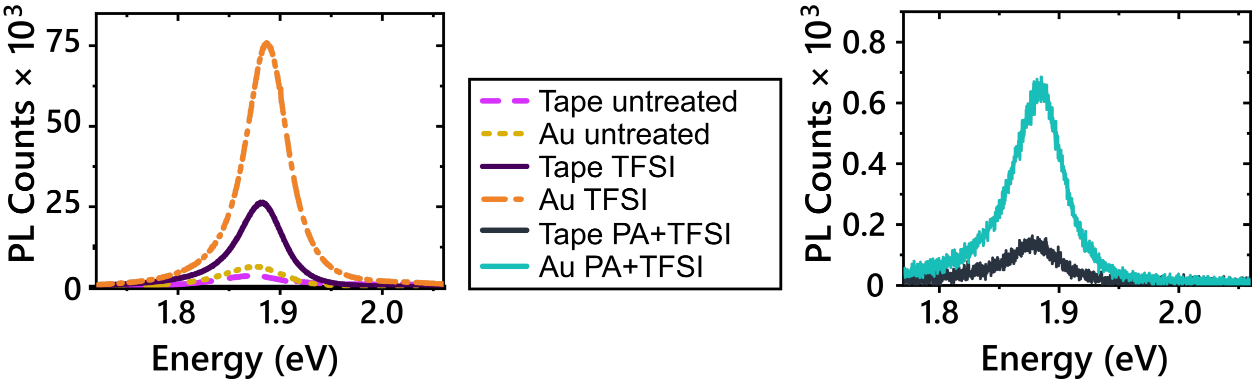

Figure 4: On the left, we plot the photoluminescence of a MoS_2 monolayer flake exfoliated using only sticky tape (tape), and using the gold exfoliation method (Au). Both the tape and Au samples exhibit a significant enhancement upon TFSI treatment. Because the PA+TFSI treated samples were even brighter and saturated the detector, we had to measure these under a higher power and different integration time, (shown on the right), in which the TFSI and untreated sample PL is undetectable. The chemical treatments show similar enhancements for the tape exfoliated samples as the gold samples.

Section 4: Subgap excitation pump-probe

For further evidence of subgap absorption, we perform pump-probe measurements exciting subgap. Theory predicts two subgap transitions. The transition from the occupied to unoccupied defect states, X_{D2}, is ∼0.2 eV lower in energy than the A exciton transition. Theory predicts a second transition, X_{D1}, from the valence band to the subgap, unoccupied defect level, which is ∼0.5 eV lower in energy than the A exciton transition. We therefore excite TFSI-treated samples at 1.67 eV and 1.38 eV (Fig 5), and both result in pump-probe spectra similar to those excited above-bandgap. In a and c, we show spectral dynamics of the TFSI-treated samples. In b and d, we plot the maximum $\Delta T/T$ at the A exciton bleach for three different powers, showing that the signal scales linearly with pump fluence and therefore is not a result of two-photon absorption. In an untreated sample, in e, even when exciting at the highest fluence of 120 $\mu J/cm^2$, the signal was nearly negligible.

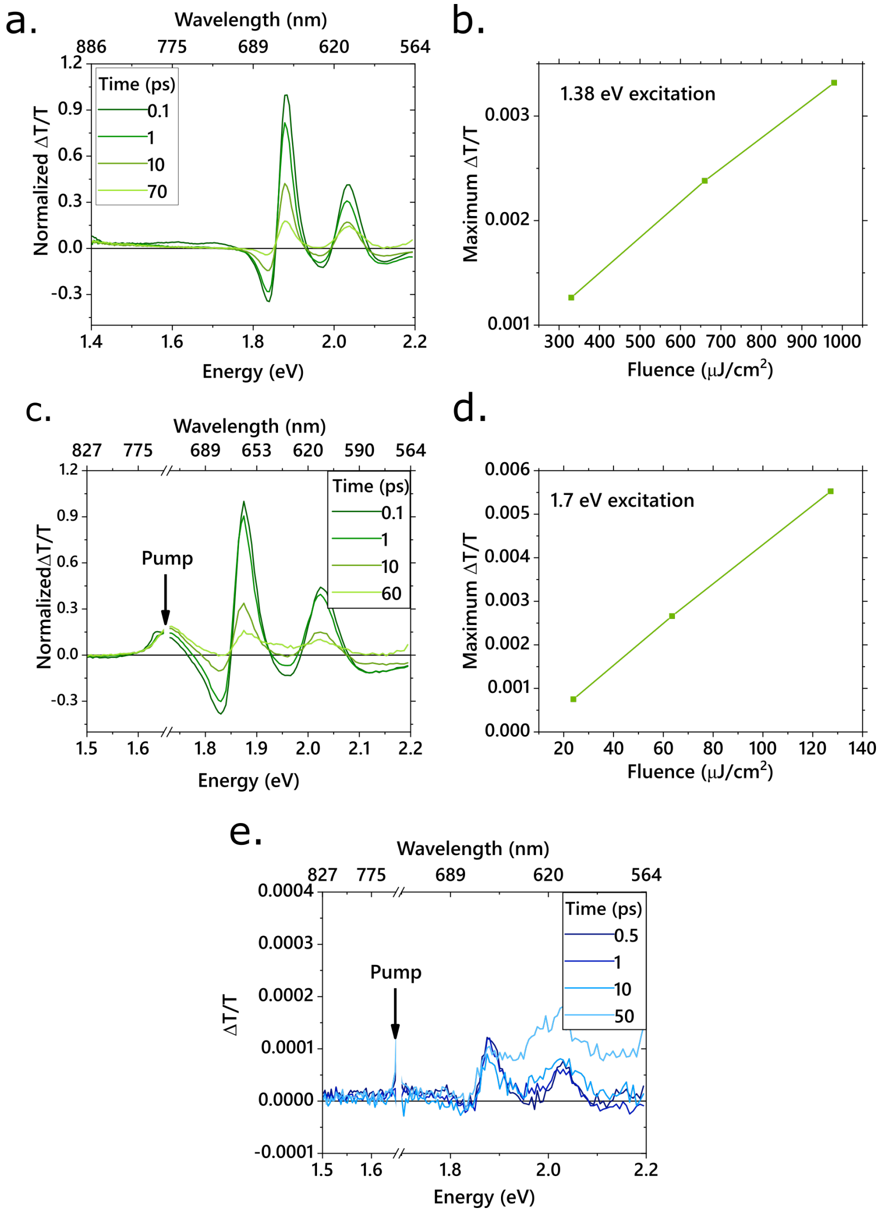

Figure 5: Pump-probe with sub-bandgap excitation. (A) Pump-probe of TFSI sample when excited at 1.7 eV with ~60 μJcm^{-2} shows characteristic pump-probe spectrum and distinct subgap bleach. b. The maximum signal taken at the A exciton bleach (~1.88 eV) for a range of pump fluences shows a linear relationship between excitation fluence and signal magnitude. (c) Pump-probe of TFSI sample when excited at ~1.38 eV with ~280 μJ/cm^2. (d) The maximum signal taken at the A exciton bleach (~1.88 eV) for a number of pump fluences shows a linear relationship between excitation fluence and signal magnitude. (e) When exciting an untreated sample at 1.7 eV and ~140 μJ/cm^2 the signal is extremely weak.

Section 5: Pump-probe on PA treated sample

When measuring samples that have been treated with only a passivating agent, no subgap state is observed (Fig. 6).

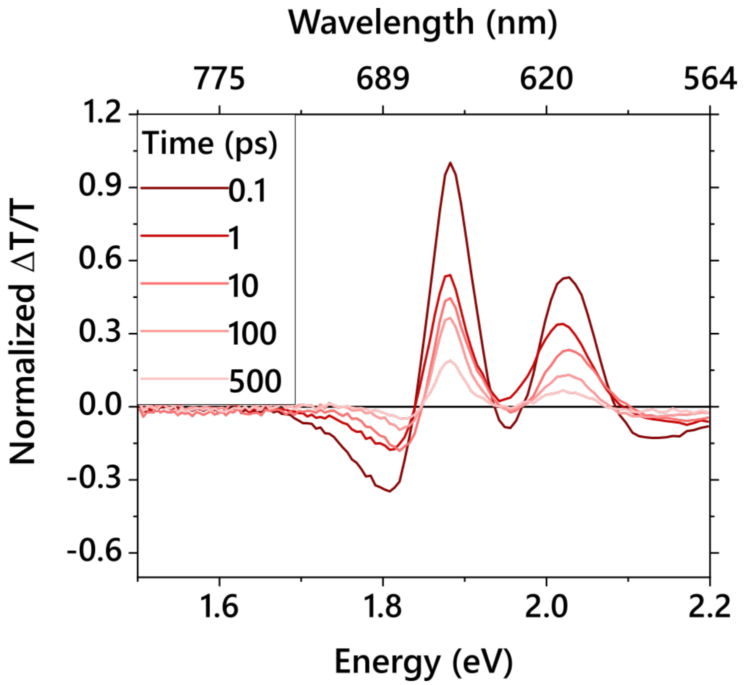

Figure 6: Pump-probe of passivating-agent only. Pump-probe measurements of passivating agent treated MoS_2 excited at 2.47 eV with $\sim$60 μJ/cm^2 shows no subgap state. Data is shown from a single sample, but has been measured on multiple monolayer flakes which display similar trends.

Section 6: Significance of treatment order

PA alone does not result in any increase in PL in MoS_2, and only a small increase in WS_2 (Fig. 7). The order of chemical treatments is significant for the enhancement of PL. A large enhancement is only observed when the passivating agent is applied first, and the superacid second. If the order is reversed, only a small increase is observed (Fig. 7).

Section 7: Untreated PL lifetime and instrument response

Untreated MoS_2 shows no significant difference from the instrument response time. We therefore conclude that its lifetime is below the instrument response time of 100 ps (Fig. 8).

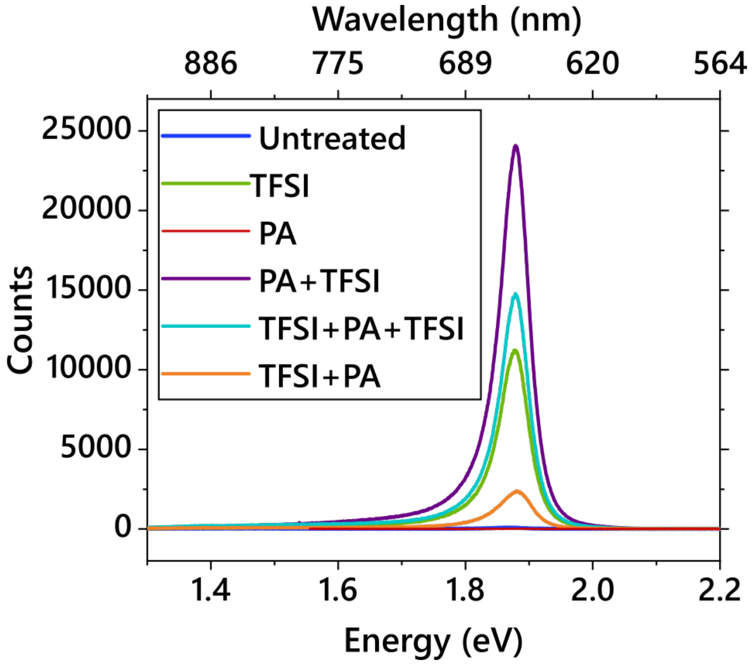

Figure 7: PL enhancement by treatment order. The passivation step must precede the TFSI to result in the largest PL enhancement. PA alone does not increase PL in MoS_2, whereas in WS_2 a small increase is observed (Fig.4b,bottom, main text). Data shown is a spectra representative of measurements taken over a number of samples, which show similar trends in magnitude of PL increase per treatment.

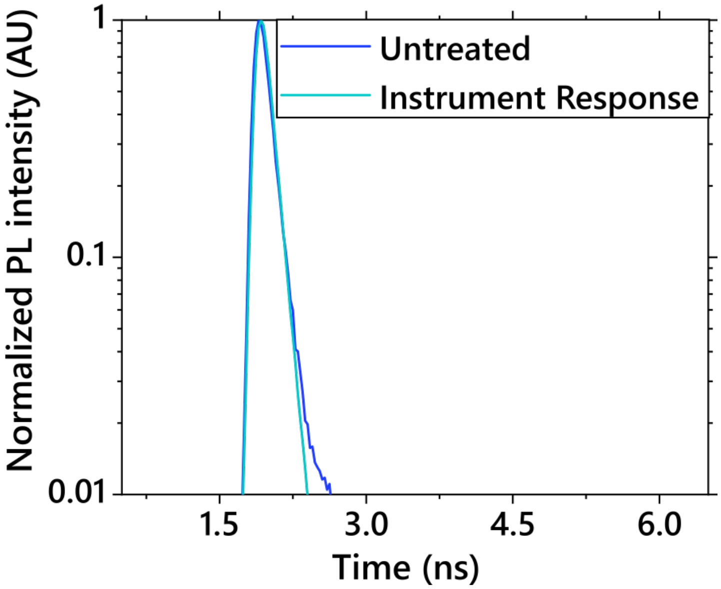

Figure 8: Instrument response of time-resolved PL measurement. Comparison of the time-resolved decay of the instrument response (green) and of the untreated sample (blue).

Section 8: Arrhenius equation and upconversion

The Arrhenius equation allows for the calculation of a rate coefficient given a temperature and activation energy. The equation is:

$$k = A\exp(-\frac{E_a}{k_B T}) \tag{2}$$

Where E_a is the activation energy, k_B is Boltzmann's constant, T, the temperature. The prefactor, A, can be given by the Raman modes of the system, in this case, A_{1g} at 408 cm^{-1} or the E_{2g} mode at 383 cm^{-1}.[1] The difference in energy between the defect state and the A exciton is $\sim$ 180 meV, however there is likely an energetic barrier to go from one state to another. We estimate the rate constant at room temperature, using the prefactor of the A_{1g} mode, and for a range of activation energies to account for possible energetic barriers.

At room temperature, the time constant for the transition from the defect state to the A exciton is on the order of picoseconds to nanoseconds. We measured a few nanosecond

Table 4: We use the Arrhenius equation to calculate the possible time constants for excitons to transition from trapped defects to the A exciton band edge given a range of energetic barriers. We assume the material is at room temperature and the known Raman modes are the prefactor.

Barrier height (meV)	Rate constant τ_U (ns)
180	0.095
225	0.320
250	0.84
300	5.8

lifetime at $8 \times 10^{-2}\mu J/cm^2$. Previous studies have measured ∼10 ns measured at $\sim 4 \times 10^{-3}\mu J/cm^2$[2] and 20 ns taken at a low fluence where exciton-exciton annihilation is not observed.[3] Given this range of 1-20 ns lifetime for radiative decay from the TFSI-treated A exciton, it is plausible that at room temperature, there is adequate energy for the band edge to be repopulated by thermal fluctuations on a timescale faster than decay from the defect states themselves. At 77 K, it has been shown that the radiative lifetime for the defect states is ∼4 μs.[3]

Section 9: GW-BSE calculation

We first performed density functional theory (DFT) calculations within the local density approximation (LDA) citekohn1965self using the Quantum-ESPRESSO code.[4] The calculations were done on a 5x5 supercell arrangement of a MoS_2 monolayer with either one S vacancy or with a single O atom substituting of one of the S atom, following prior work.[5] We used a plane-wave basis and norm-conserving pseudopotentials with a 125 Ry plane-wave cutoff. We included the Mo semi-core 4d, 4p, 4s, 5s and 5p states as valence states in our pseudopotential. The distance between repeated supercells in the out-of-plane direction was 15 Å. We relaxed the geometry within DFT and used DFT charge density as a starting point for our GW and Bethe Salpeter equation (GW-BSE) calculations.

Our GW calculations are performed with the BerkeleyGW code[6] using the generalised

plasmon-pole model,[7] an energy cutoff of 25 Ry for the planewave components of the dielectric matrix, and a nonuniform k-point sampling corresponding to a 30x30 uniform k-grid.[8] We included 4000 unoccupied states in the sum over empty states in the self energy and polarizability and tested the convergence with respect to unoccupied states by comparing to a calculation using up to 20,000 effective states obtained by averaging high-energy states within a small energy window. We find that these parameters converge the relative quasiparticle energies for the defect and pristine states at the K point to within 100 meV.

The BSE was also solved with the BerkeleyGW code.[6,9] We diagonalise the BSE Hamiltonian within the Tamm-Dancoff approximation with 10 valence and 10 conduction bands. The clustered sampling interpolation (CSI) scheme[8] was used to interpolate a nonuniform k point sampling to a uniform k-grid of 18x18x1 k points. A 5 Ry cutoff was used for the planewave components of the dielectric matrix used in the BSE. Spin orbit coupling was added as a perturbation. We find that these parameters converge the calculated exciton excitation energies up to 200 meV.

Discussion of substitutional oxygen and sulfur vacancies

Based on these results and previous literature, we propose two possible reasons that the PLQY can be increased by TFSI-treatment. First, in untreated MoS_2, n-doping may result in the predominance of trions which quench the PL emission.[10–12] When TFSI, functioning as a Lewis acid, is applied on the surface of MoS_2, the excess electrons of MoS_2 are removed, leading to a shift in threshold voltage observed in transistors as well as the blueshift in the PL. As the samples become more intrinsic, neutral excitons dominate leading to an increase in PL. Another possible explanation is that oxygen substitutions are the dominant defect, rather than sulfur vacancies, and that these create local sites with metallic character, providing a route for non-radiative recombination that quenches the PL. Given that the samples are known to be n-doped,[10,13–15] it is possible that both oxygen substitutions, and

n-doping, both reduce the overall PLQY.

The n-doping explanation (as the dominant factor contributing to a low QY) is not fully satisfactory given our experiments and the current literature. First, a number of studies report significant reductions in n-doping and large Fermi-level shift, but observe only modest increases in PL.[16–18] We have similarly found that molecular p-dopants lead to modest gains in PLQY. If the Fermi-level shift is as significant and the low PL is predominantly caused by n-doping, we would expect molecular dopants to see a large increase in PL. Second, we would also expect that molecular doping may result in a subgap absorption state, but this has not been observed.

However, we have struggled to find any evidence of the presence of oxygen substitutions. Indeed the identification of oxygen substitutions is extremely challenging and requires the use of UHV based techniques like STM, which are not compatible with the wet chemical passivation methods used here. Thus, our experimental results and their close correspondence with theory predictions supports the idea that the TFSI treatment results in a sulfur vacancy, and the PA+TFSI treatment passivates this. However, the nature of these sulfur vacancies in untreated materials, if they are filled with oxygen or n-doping will need future experimental and theoretical study.

References

(1) Lee, C.; Yan, H.; Brus, L. E.; Heinz, T. F.; Hone, J.; Ryu, S. Anomalous Lattice Vibrations of Single-and Few-Layer MoS_2. *ACS Nano* **2010**, *4*, 2695–2700.

(2) Amani, M. et al. Near-Unity Photoluminescence Quantum Yield in MoS_2. *Science* **2015**, *350*, 1065–1068.

(3) Goodman, A. J.; Willard, A. P.; Tisdale, W. A. Exciton Trapping is Responsible for the Long Apparent lifetime in Acid-Treated MoS_2. *Physical Review B* **2017**, *96*, 121404.

(4) Giannozzi, P. et al. QUANTUM ESPRESSO: A Modular and Open-Source Software Project for Quantum Simulations of Materials. *Journal of Physics: Condensed Matter* **2009**, *21*, 395502.

(5) Refaely-Abramson, S.; Qiu, D. Y.; Louie, S. G.; Neaton, J. B. Defect-Induced Modification of Low-Lying Excitons and Valley Selectivity in Monolayer Transition Metal Dichalcogenides. *Physical Review Letters* **2018**, *121*, 167402.

(6) Deslippe, J.; Samsonidze, G.; Strubbe, D. A.; Jain, M.; Cohen, M. L.; Louie, S. G. BerkeleyGW: A Massively Parallel Computer Package for the Calculation of the Quasiparticle and Optical Properties of Materials and Nanostructures. *Computer Physics Communications* **2012**, *183*, 1269–1289.

(7) Hybertsen, M. S.; Louie, S. G. First-Principles Theory of Quasiparticles: Calculation of Band Gaps in Semiconductors and Insulators. *Physical Review Letters* **1985**, *55*, 1418.

(8) Felipe, H.; Qiu, D. Y.; Louie, S. G. Nonuniform Sampling Schemes of the Brillouin Zone for Many-Electron Perturbation-Theory Calculations in Reduced Dimensionality. *Physical Review B* **2017**, *95*, 035109.

(9) Rohlfing, M.; Louie, S. G. Electron-Hole Excitations and Optical Spectra from First Principles. *Physical Review B* **2000**, *62*, 4927.

(10) Pető, J.; Ollár, T.; Vancsó, P.; Popov, Z. I.; Magda, G. Z.; Dobrik, G.; Hwang, C.; Sorokin, P. B.; Tapasztó, L. Spontaneous Doping of the Basal Plane of MoS_2 Single Layers through Oxygen Substitution under Ambient Conditions. *Nature chemistry* **2018**, *10*, 1246–1251.

(11) Lien, D.-H.; Uddin, S. Z.; Yeh, M.; Amani, M.; Kim, H.; Ager, J. W.; Yablonovitch, E.; Javey, A. Electrical Suppression of All Nonradiative Recombination Pathways in Monolayer Semiconductors. *Science* **2019**, *364*, 468–471.

(12) Mak, K. F.; He, K.; Lee, C.; Lee, G. H.; Hone, J.; Heinz, T. F.; Shan, J. Tightly Bound Trions in Monolayer MoS_2. *Nature materials* **2013**, *12*, 207–211.

(13) Chiu, M.-H.; Zhang, C.; Shiu, H.-W.; Chuu, C.-P.; Chen, C.-H.; Chang, C.-Y. S.; Chen, C.-H.; Chou, M.-Y.; Shih, C.-K.; Li, L.-J. Determination of Band Alignment in the Single-Layer MoS_2/WSe_2 Heterojunction. *Nature communications* **2015**, *6*, 1–6.

(14) McDonnell, S.; Addou, R.; Buie, C.; Wallace, R. M.; Hinkle, C. L. Defect-Dominated Doping and Contact Resistance in MoS_2. *ACS Nano* **2014**, *8*, 2880–2888.

(15) Bruix, A.; Miwa, J. A.; Hauptmann, N.; Wegner, D.; Ulstrup, S.; Grønborg, S. S.; Sanders, C. E.; Dendzik, M.; Čabo, A. G.; Bianchi, M.; Lauritsen, A. A., Jeppe V.and Khajetoorians; Hammer, B.; Hofmann, P. Single-Layer MoS_2 on Au (111): Band Gap Renormalization and Substrate Interaction. *Physical Review B* **2016**, *93*, 165422.

(16) Mouri, S.; Miyauchi, Y.; Matsuda, K. Tunable Photoluminescence of Monolayer MoS_2 *via* Chemical Doping. *Nano Letters* **2013**, *13*, 5944–5948.

(17) Kim, Y.; Lee, Y.; Kim, H.; Roy, S.; Kim, J. Near-Field Exciton Imaging of Chemically Treated MoS_2 Monolayers. *Nanoscale* **2018**, *10*, 8851–8858.

(18) Zhang, S.; Hill, H. M.; Moudgil, K.; Richter, C. A.; Hight Walker, A. R.; Barlow, S.; Marder, S. R.; Hacker, C. A.; Pookpanratana, S. J. Controllable, Wide-Ranging *n*-

Doping and *p*-Doping of Monolayer Group 6 Transition-Metal Disulfides and Diselenides. *Advanced Materials* **2018**, *30*, 1802991.
